# Chondrosarcoma of Mandible: A Rare Case Report

**DOI:** 10.22038/IJORL.2021.47251.2553

**Published:** 2021-11

**Authors:** Vanita Sarin, Karanvir Singh, Vikas Kakkar, Gursimranjitt Singh

**Affiliations:** 1 *Department of Otorhinolaryngology, * *Shri Guru Ram Das Institute of Medical Sciences and Research, * *Amritsar, India.*; 2 *Department of Surgery, * *Shri Guru Ram Das Institute of Medical Sciences and Research* *, Amritsar, India.*; 3 *Department of Plastic Surgery, * *Shri Guru Ram Das Institute of Medical Sciences and Research* *, Amritsar, India.*

**Keywords:** Chondrosarcoma, Malignant, Mesenchymal, Neoplasm, Mandible

## Abstract

**Introduction:**

Chondrosarcomas (CS) are malignant mesenchymal tumours with cartilaginous differentiation that rarely affects the maxillofacial region. It accounts for approximately 10-20% of malignant bone tumours. The CS is rare in occurrence with aggressive course with high malignant potential, and poor prognosis.

**Case Report:**

Here we report a rare case of a 37year old female presenting tumoral mass in the lower jaw, for the past 3 years, which was gradually progressive in nature with an area of skin ulceration. The CT revealed a well- defined lesion with soft tissue component measuring 15x12x10 cm in size infiltrating both right and left masseter. Biopsy confirmed the diagnosis of grade IIICS. Considering the size and aggressive nature of the lesion, surgical resection was done. The reconstruction of the mandible was done with vascularised fibula pedicle flap to achieve acceptable cosmesis. The patient was discharged uneventfully. Though few cases of high grade CS have been reported in literature but CS of this enormous size has not been reported yet.

**Conclusion:**

In this case report we documented the management of a relatively rare but challenging reconstructive maxillofacial surgery. Since in this case the CS was enormous in size and aggressive surgery was required, the cosmetic and functional outcomes were challenging. In our case report we have found that a vascularised fibula pedicle flap gives a good functional and cosmetic outcome and can be used for reconstruction of complete mandible.

## Introduction

CS are malignant mesenchymal tumours with cartilaginous differentiation that rarely affects the head and neck region arising in long and flat bones.^1^ Around 10-20% of malignant bone tumours and about 0.1% of all head and neck neoplasms has been reported in literature^1.^ Majority of CS of head and neck arise from maxilla, with very few cases arising from mandible with a high rate of occurence. The World Health Organization (WHO) classified CS “as a malignant tumour with pure hyaline cartilage differentiation characterized by the formation of cartilage(1), but not of bone, by tumour cells”(2). 

The pathogenesis and biologic behavior of this chondrogenic tumor is poorly understood, but it is evident that these lesions represent a spectrum from benign chondroma to the malignant CS, through varying degrees of intermediate type (3). Age of presentation is between ages 30-60 years (4). Patients typically present with vague symptoms of pain and swelling that may have been present for a relatively long duration (3).

Histology is the gold standard to the diagnosis, characterized by a biphasicpattern consisting of areas of hyaline cartilage mixed with small cell malignancy (5).In head and neck region. CS of Grade I are frequently reported. The present case was diagnosed as Grade III CS, enormous in size, involving whole of the mandible, the management of which makes this more important to discuss and document.

## Case Report

A 37 year old female patient presented in ENT outpatient department with chief complaints of tumoral mass in lower jaw since 3 years and decreased mouth opening since 1 year. The swelling was gradually progressive in nature with an area of skin ulceration of approximately around 4x4 cm at the inferior aspect of the swelling with bleeding and foul smelling debris within. On examination swelling was 16x14 cm in size, irregular shape fixed to the jaw, extending from left pre-auricular region to right pre-auricular region and vertically around 16 cm long. 

On palpation, the inspection findings were confirmed, the swelling was immobile, hard in consistency and slightly tender. The neck mobility was restricted because of its enormous size but the jaw mobility was not restricted and the mouth opening of the patient was two finger breadths. The skin overlying the ulcerated area was adherent while the rest was non adherent without infiltration but showed dilated veins. In the intra-oral examination the swelling extended intra-orally obliterating both the buccogingival and tonsilo-lingual sulcus from right ramus to left ramus but bilateral temporomandibular joints were spared.

**Fig 1:**
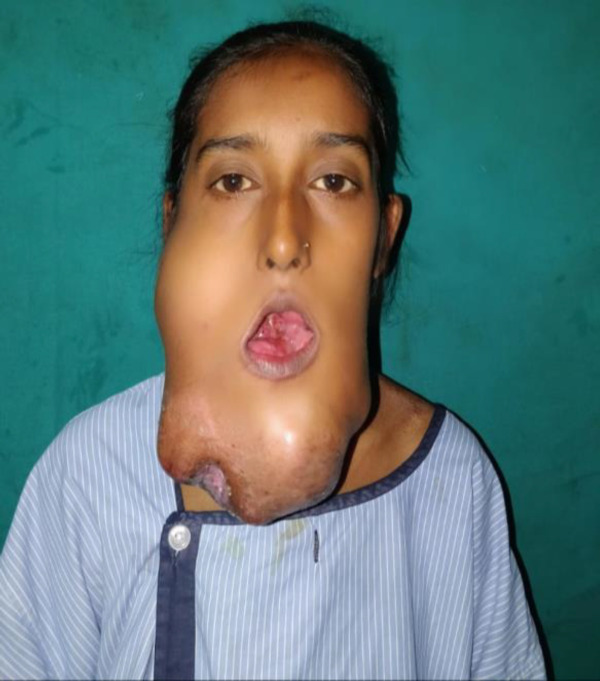
Clinical Pre-Operative showing 16*14cm jaw lesion, irregular shape with an ulcerated area of 4*4mm at inferior aspect

 There was no ulceration of the intraoral mucosa. There was grade 2 loosening of teeth.Tongue was free of the lesion. There was no significant palpable lymphadenopathy (Fig 1).

Computed Tomography (CT) scans revealed a large relatively well defined soft tissue density lesion with amorphous, ring and arch like calcifications seen in relation to mandibular bone with erosion and destruction of its body, alveolar margin and bilateral rami. 

The soft tissue component measured 15x12x10 cm in size. The lesion was involving the right masticator space with the infiltration of the right masseter. 

**Fig 2:**
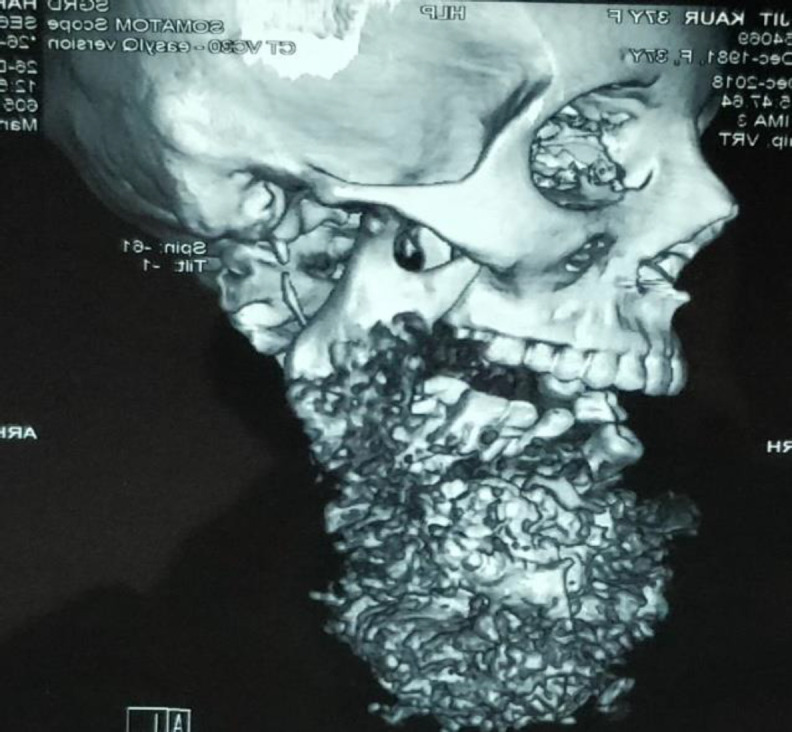
3D CT (Right) reveals soft tissue infra-condylar lesion 15*12*10cm

Infiltration into the left masticator space was also noted with the involvement of the left masseter muscle. Encroachment and compression of the sublingual space was also seen (Fig 2).

**Fig 3:**
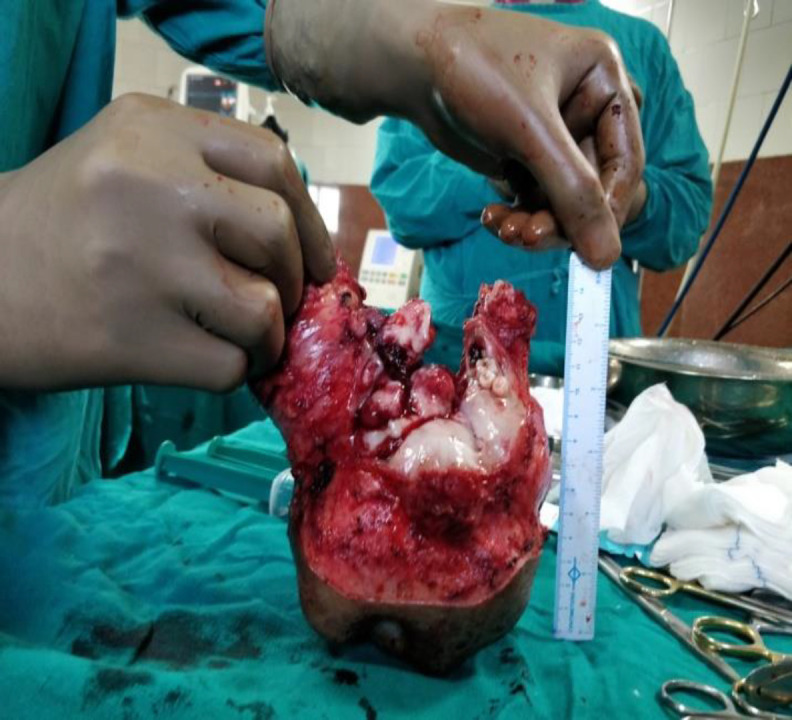
Specimen of Mandibulectomy with Wide Margins

Microscopic examination of the incisional biopsy revealed chondrocytes and lacunae in lobular patterns with pleomorphic and hyperchromatic nuclei. Mitosis was seen. A prominent cellular spindle cell proliferation was seen with minimal areas of chondroid. A high grade III CS was confirmed. The general workup of the patient revealed no distant metastasis. The surgical resection was executed based on the size and aggressiveness of lesion. The prognosis and management was explained to the patient and after taking consent, a mandibulectomy with wide margins of the lesion leaving bilateral condyloid processes of the mandible was done (Fig 3). 

**Fig 4:**
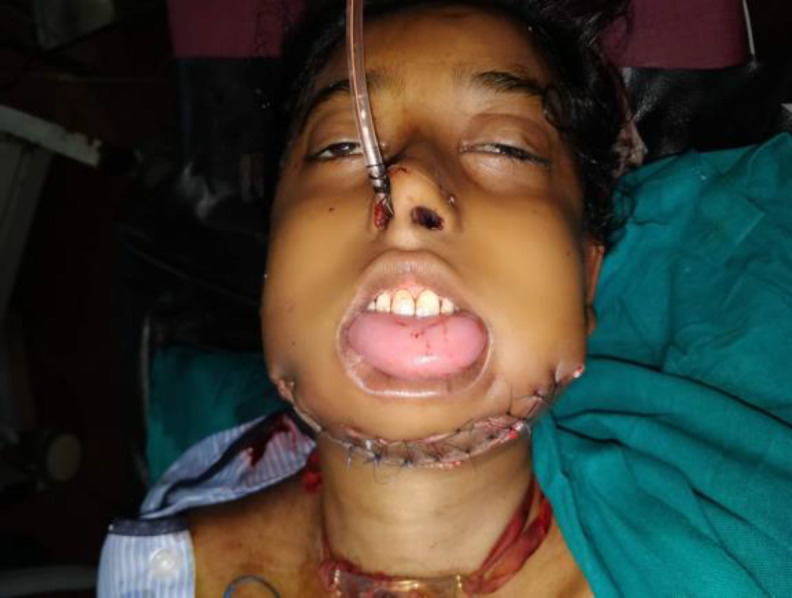
Post-Operative Clinical picture of patient with reconstruction of mandible with vascularized fibula pedicle flap and tracheostomy

The reconstruction of the mandible was done with free vascularised fibula flap based on peroneal artery and it`s perforators. The arterio-venous anastomosis was done with the facial vessels. Titanium plates with screws were used for giving shape to the fibula as a jaw bone. The patient was tracheostomised at the end of the procedure, successfully extubated and shifted to ICU uneventfully (Fig 4). 

The pathological specimen sent post operatively, showed chondroid tissue along with myxoid tissue. The chondroid tissue at periphery show presence of sheets of tumour cells separated by thick fibrous collagenous stroma. The cells are ovoid to spindly, hyperchromatic, pleomorphic nuclei with prominent nucleoli in some. Mitotic activity also increased at places along with small areas of necrosis (Fig5.6).

**Fig 5:**
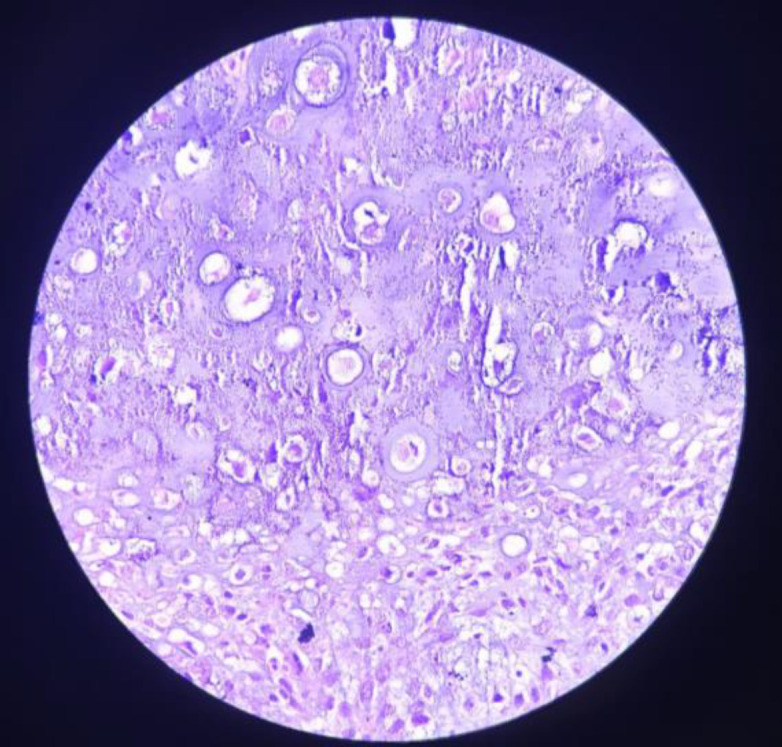
HPE 40X chondroid tissue at periphery shows presence of sheets of tumor cells separated by thick fibrous collagenous stroma with increased mitotic activity

**Fig 6:**
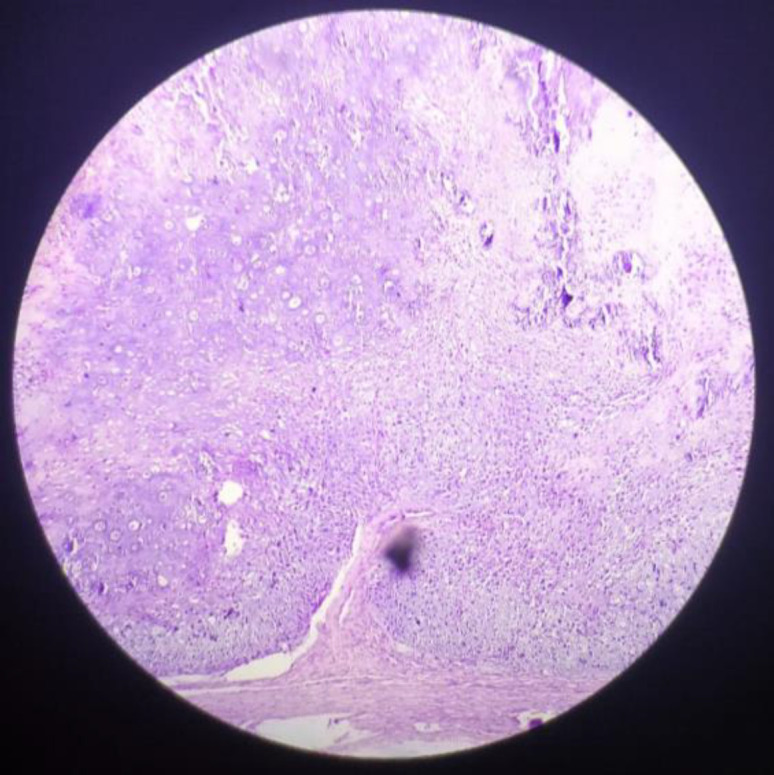
HPE 10X showing chondroid tissue along with myxoid tissue with increased mitotic activity and areas of necrosis

The bony and soft tissue surgical margins were free of tumour. Immuno-histochemistry revealed strong expression of KI-67, thus confirming the diagnosis of high grade CS. The patient was discharged uneventfully on a tracheostomy tube on 18^th^post operative day, and was further advised for radiotherapy and chemotherapy. The patient was receiving further treatment when she met with a road accident and died.

## Discussion

CS is rare in the head and neck region which accounts for 4.2-6.7% of cases. Most CS of the head and neck region occur in the maxilla; others are found in descending order of frequency in the body of the mandible, ramus, nasal septum and the paranasal sinuses(6,7).CS arising from mandible is very rare and occurs mostly in the mandibular symphysis region.The present case occurred in the mandible.

CS are malignant tumors arising from cartilage cells that tend to maintain their essentially cartilaginous nature throughout their evolution. These neoplasms mostly occur in the long bones, pelvis and ribs (1). Though the pathogenesis arise de novo whereas secondary CS are thought to arise from pre-existing lesions like Ollier`s disease, solitary osteochondroma, Maffucci syndrome, solitary enchondroma, Paget`s disease or radiation injury. It is now believed that cell cycle regulators p16, p53 and retinoblastoma play important roles in the development of this tumor (8).

 The most common clinical finding is a painless swelling, expansion of the buccal and the lingual plates, premature eruption or exfoliation of teeth. The mass is usually rapidly growing and covered with mucosa which can ulcerate followed by pain at later stages (9). These features were consistent with the present case.Diagnosis can only be established by combination of clinical, radiographic and histopathological examination.

There are no radiographic findings that are pathognomonic for CS.The radiological pattern of CS is variable. These lesions may appear radiographically as osteolytic, with radiolucent shadows with a wide zone of transition and irregular or indistinct borders. Alternatively, they may demonstrate an ill-defined cloud-like matrix with the calcified “whorls and arcs” typical of a chondroid matrix plus or minus aggressive features such as endosteal scalloping, cortical disruption, periostitis, and or soft tissue mass effect (10). These lytic changes are prominent in more advanced cases. Furthermore, it may reveal a ground glass appearance or a sunburst appearance. In this case an enormous and aggressive soft tissue density lesion with amorphous, ring and arch like calcifications, erosion and destruction of body of mandible, alveolar margin and bilateral rami was seen.

Histopathology is mainstay of diagnosis. Grade 1 CS closely mimic the appearance of a chondroma, composed of a chondroid matrix and chondroblasts that show only a subtle variation from the appearance of normal cartilage. Calcification or ossification of the cartilaginous matrix is often prominent and mitosis being a rare feature. Grade II CSs show a greater proportion of moderately sized nuclei and increased cellularity. The cartilaginous matrix tends to be more myxoid. The mitotic rate is low. Grade III CSs are highly cellular and show spindle cell proliferation with mitosis. Easily recognizable cartilage may be rare with a higher proliferative index (11-13), this was confirmed in our case by the use of proliferative marker Ki-67 which showed strong expression.Few cases of high grade CS have been reported in literature but CS of this enormous size and aggressiveness has not been reported yet. This makes this case report all the more interesting. Wide surgical excision with 2-3 cm healthy margins was done and to achieve this we had to remove the whole of the mandible sparing the condyloid process of both sides. Removal of 15cmx12cmx10cm sized lesion with 2-3 cm of adequate healthy margins created a huge defect which posed as a challenge for the surgeon, because ideal reconstructive method should provide not only satisfactory structural cosmesis, but also good restoration of function. So the jaw reconstruction was aided by free vascularised fibula flap, due to the ease to tailor the flap precisely to the defect which provided reconstruction of not only the shape but a functional mandible.

Because of the rarity of CS of the jaw there are no established evidence-based treatment protocols. For this reason, many of the current treatment strategies for this disease have been extrapolated from protocols developed and tested for CS of other more typical locations outside the head and neck region or other types of sarcomas. The most effective therapeutic modality is wide surgical excision (14). Wide local excision with a tumour free margin of 2-3cm is recommended. Questions persist, however, regarding the use and timing of chemotherapy. Most surgeons would agree on neo-adjuvant chemotherapy in cases of CS, because it is intuitive that clear margins would otherwise be unobtainable without undue morbidity. Although combined chemotherapy did not decrease the size of the tumour, it inhibited tumour growth and spread (15). The use of chemotherapy postoperatively is also controversial in the case of clear pathological margins. Of course, information such as tumour distance from the margins and histological grading is useful in the decision-making process (16).Although, in general, CS have been thought to be resistant to chemotherapy, a great interest has emerged about its use in metastatic disease and in dedifferentiated forms. There is possible role of chemotherapy for the de differentiated forms; the dedifferentiated component is a high grade spindle and/or pleomorphic sarcoma, and a positive response has been documented for other high grade sarcomas treated with chemotherapy (17). Also, the postoperative radiotherapy and chemotherapy offer a good prognosis and eradicate chances of micrometastases. However wide surgical resection is the preferred choice of treatment as CS are traditionally radio-resistant (18). But, radiotherapy can be opted as an adjunct for high-grade lesions (18). In this case too though adequate surgical margins were achieved per-operatively but considering that the lesion was high grade and the postoperative histopathology revealed that the tumour distance from the surgical margins was not adequate in the right superior margin, so post-operative chemo-radiation was mandatory.

The main prognostic factors are surgical resection, stage, grade and primary site. The mesenchymal and dedifferentiated forms are known to have poor prognosis. 

The prognosis is poor because tumours have a tendency for early recurrence either locally or as metastasis. Metastasis is hematogenous and the most common site is the lung (14). The prognosis of CS of the jaw is poor compared to any other part of the body because of the difficulty to achieve tumor free surgical margins. 

Five-year survival is about 90% for grade 1 CS and around 50% for combined grade II and III CS. Occasionally, CS show the coexistence of various histological grades in the same tumour (19). In a study conducted by AC Camargo Hospital, Prado et al. observed recurrence in 4 of 10 previously treated patients. This figure is similar with other studies. Arlen et al. described 10 recurrences out 18 treated patients (20), and Mark et al. showed a recurrence rate of 44% (21). Fatality is mainly due to extension into the base of the skull or distant metastasis usually to lungs and bones (18). The prognosis is good for low and intermediate grade CS. The recurrence of head and neck CS is frequent because of the complicated location preventing complete surgical excision of the lesion (22).

## Conclusion

Though cases of grade III CS have been reported in literature, but CS of this enormous size and aggressiveness has not been reported yet. 

Because of the rarity of CS of the jaw there are no established evidence-based treatment protocols. The most effective therapeutic modality is wide surgical excision. We recommend radical approach for resection of such giant and aggressive lesions and ideal reconstructive methods which provide both aesthetic and functional results as we did in this case by a free vascularised fibula flap, due to the ease to tailor the flap precisely to the defect which provided not only reconstruction of the shape but a functional mandible.
